# Strategically growing the urban forest will improve our world

**DOI:** 10.1038/s41467-018-03622-0

**Published:** 2018-03-21

**Authors:** Theodore A. Endreny

**Affiliations:** Department of Environmental Resources Engineering, SUNY ESF, Syracuse, NY 13210 USA

## Abstract

Growth in urban populations creates opportunities for urban forests to deliver ecosystem services critical to human wellbeing and biodiversity. Our challenge is to strategically expand urban forests and provide our international communities, particularly the vulnerable, with healthier, happier, and enriched lives.

Trees are too often removed for urbanization, well captured by Joni Mitchell’s lyrics “They paved paradise. And put up a parking lot.” Urban areas globally will expand to accommodate population growth and migration trends^1^. Yet, urban denizens benefit greatly with trees in their habitat, and that is the theme of the 2018 International Day of Forests; Forests and Sustainable Cities. Urban areas can concentrate poverty and sickness, and trees can help alleviate these ills through their ecosystem services. Our global challenge is to grow urban forests and sustain human wellbeing and biodiversity.

The urban forest is defined to comprise all trees in the urban area, inclusive of individual street trees and clusters of park trees, and peri-urban forests extend to the outer metropolitan area. Within the urban forest, forest types include city parks and urban forests >0.5 ha, pocket parks and gardens with trees, trees on streets or in public squares, and any other green spaces with trees, such as riparian corridors, rooftops, and nurseries. Urban areas occupy 4% of the world’s land area, and if planted at global average tree density, they could contain 121 billion trees^2^. Urban forests may have <10 billion trees, with >100 genus including *Pinus*, *Platanus*, and *Pyrus* that are increasingly planted based on the ecosystem services they can deliver.

## Establishing the role of ecosystem services

Ecosystem services from trees can be categorized as cultural (e.g., spiritual, recreational), provisioning (e.g., food, fiber, water), regulating (e.g., climate and flood control), and supporting (e.g., pollination, soil formation). Causation between tree structure and functional services include the obvious, such as harvesting an apple, to processes that only researchers may notice such as cooling buildings through shading, cooling the air through transpiration, silencing of noise through damping, and cleaning the air and water through filtration. In general, ecosystem services are greater from evergreen trees with large leaf areas due to the functional role of tree canopy^3^. The Food and Agriculture Organization^4^ mapped how urban forest advance nine UN Sustainable Development Goals (SDGs): no poverty, zero hunger, good health and well-being, clean water and sanitation, affordable and clean energy, descent work and economic growth, climate action, life on land, and sustainable cities and communities (see Table 1). Preliminary analysis suggests an average return on investment of $2.25 for each $1 invested in urban trees, and this >100% return does not include all services^5^. Urban forest services are invaluable for the vulnerable and low capacity residents without food, water, and energy security, who can find in these forests nutrition, clean water, wood fuel, and shelter, as well as jobs and a sense of purpose.

**Table 1:** Services from five urban forest types advance UN Sustainable Development Goals with varying significance

Urban forest service	SDGs^a^	Significance of contribution by urban forest type (5 = very high, 1 = very low)				
		Peri-urban forest and woodlands	City parks and urban forests > 0.5 ha	Pocket parks and gardens with trees	Trees on streets or in public squares	Other green spaces with trees
Human health and well-being	3, 11	5	5	5	5	5
Climate change mitigation	11, 13	5	3	1	1	2
Climate change adaptation	11, 13	5	5	4	4	4
Biodiversity and landscapes	11, 15	5	4	3	2	3
Economic benefits and green economy	1, 2, 7, 8, 11	5	4	3	3	1
Land and soil degradation	11, 13, 15	5	3	2	1	4
Watershed protection	2, 6	5	2	n.a.	1	4
Resilience to flooding events	2, 6	3	5	3	3	4
Food and nutrition security	2, 11	5	3	2	2	4
Wood security	2, 11	5	2	1	1	2
Recreation	11	4	5	3	1	3
Education	11	5	4	1	1	3
Social cohesion	11	2	5	3	1	2
Social security and equity	11	1	4	4	5	3

^a^#1 = no poverty, #2 = zero hunger, #3 = good health and well-being, #6 = clean water and sanitation, #7 = affordable and clean energy, #8 = descent work and economic growth, #11 = sustainable cities and communities, #13 = climate action, #15 = life on land

Healthcare, education, and military spending typically consume the largest fraction of governmental budgets, and trees assist with disease prevention, therapy, and recovery, making for a healthier and happier population^6,7^. Urban forests can contribute to a tenth SDG of peace, just and strong institutions by providing shared spaces that enhance mixing of community across ages, cultures, and incomes^4^. Evidence from urban forests suggests they contribute to an 11th SDG of industry, innovation, and infrastructure, generating $148 billion annually in the US from arboriculture and landscape design expenditures^5^. London’s urban forest was estimated to have 8,421,000 trees yielding annual benefits of £132.7 million (not including all services), a replacement cost of £6.12 billion, and an amenity value of £43.3 billion^8^. The London study was extrapolated to the megacities of Beijing, Buenos Aires, Cairo, Istanbul, Los Angeles, Mexico City, Moscow, Mumbai, and Tokyo and estimated the annual benefit of forests within these metropolitan areas had a median annual value of $505 million, the potential to obtain nearly $1 billion in annual benefits, and an additional $7.9 billion in total value of carbon storage^9^. With money effectively growing on trees, what could slow growth of the urban forest?

## Roadblocks to improving urban forests, existing and future

Barriers to advancing the urban forests include actual, potential, and perceived tree disservices, which are outnumbered by services^10^. Disservices can vary with income, culture, and environment, making them local phenomena. Financial costs disservices of urban trees arise with planting and management, injury to humans and infrastructure due to accidents, trapping of air pollutants beneath the canopy, and additional winter heating of buildings due to tree shade. Social disservices of trees arise when generating allergens, obstructing views and passage, harboring pests and disease vectors, and engendering fear of crime or injury; the risk of being killed by a falling tree limb is one in ten million^4^. Environmental disservices arise with the misuse of chemicals or power tools in tree maintenance, release of greenhouse gases in tree decomposition, increase of harmful ozone from tree biogenic volatile organic compounds (BVOC), and displacement of native species with exotic tree plantings. Globally, barriers to advancing urban forests are emerging with climate disruption and land cover changes, creating indirect roadblocks when natural disaster recovery consumes urban forest budgets, and direct roadblocks when trees are lost due to extreme temperatures, wildfire, sea-level rise, flooding, drought, salinization of soils, and invasive pathogens and pests, the latter killing more trees as urban areas warm^11^. Insidiously, more atmospheric CO_2_, pollution, and higher air temperatures are shown to trigger more powerful pollen allergens and BVOC emissions^3^. Yet where there are threats, there are opportunities.

## Opportunities to improve urban forests

A virtuous cycle is possible for extending urban forests, with benefits paying for management, and new forests advancing research to maximize services and minimize disservices. The field of urban forestry will grow with that of urban science, which is poised to grow rapidly, generating discoveries at the social-ecological system nexus critical to sustainability^12^. Linking urban forestry to ecological engineering provides an opportunity to focus on building with nature to achieve renewably powered and systems-based self-designs that satisfy human needs and advance ecosystem conservation. Given the audacity of designing with ecosystems, this field embraces the adaptive management strategy of learning from failure. Innovations include new vertical forests overlaying multi-story buildings, utilizing vertical space to best service building occupants and to capture pollutants^6^. Partnering urban forestry with eco-technologies can benefit business and provide services critical for human well-being yet beyond the capacity of trees. Opportunities exist to integrate urban forestry with high-profile, relatively well-funded measures, including restoration of watersheds, wetlands, and coastal systems^13^, and reworking urban form when engaged in the disruptive process of replacing gray infrastructure with green infrastructure^14^. Abnormal opportunities have emerged with anthropogenic disruptions to climate and biogeochemical cycles; global warming has triggered longer growing seasons, and elevated fertilization and atmospheric CO_2_ have accelerated tree growth, possibly leading to larger trees with greater ecosystem services^15^. Exciting discoveries will emerge in urban forestry as we manage these threats and pursue these opportunities.

## Knowledge gaps and the way forward

Urban forestry is a transdisciplinary field involving the entire community, with its members and their interactions contributing to the many unknowns in formulating holistic policy, science, and management for sustainable cities. Research needs for advancing urban forestry have been summarized by the FAO^4^, and include environmental, social, and economic aspects, at a range of spatial and temporal scales. The environmental unknowns include establishing tree characteristics and planting opportunities, selecting tree species to increase diversity, service delivery, and forest resilience^3^, discovering the role of the microbiome in tree health, managing for biotic and abiotic stresses such as pests, fire, and pollution, and linking urban forests and human health, with the latter connecting to social and economic research such as public−private partnerships in the payment for ecosystem services. Formal and informal education programs at all age levels are structured around these knowledge gaps, encouraging citizen science, enriching inquiry-based learning, and empowering citizens to speak on behalf of their trees.

The way forward for urban forestry is to design landscapes in partnership with the UN SDGs and uphold an international commitment to improving human wellbeing and biodiversity. Urban forest design tools include i-Tree (www.itreetools.org), which enable a leaderless movement where citizens can inventory their urban forests, discover the benefits of different tree species, select optimal planting locations and tree size distributions, and determine how trees clean their air and water, mitigate climate change, and many other services. The urban forest movement will benefit from holistic policy and legal frameworks, strategic planning and design, and adaptive management, monitoring and financing for humans and nature. We need to tell the stories of success from communities across the globe, where fruit harvests supply food banks in Seattle USA, greenbelts treat wastewater and combat desertification in Ouarzazate, Morocco, toxic soils are cleansed by trees in Guangxi, China, urban temperatures and poverty are reduced with forest stands in Bobo-Dioulasso, Burkina Faso, tree nurseries purify drinking water and generate wood fuel for the needy in Dhaka, Bangladesh; and children in an impoverished school are nurtured by a tree garden irrigated with gray-water within the desert landscape of Lima, Peru^4^. These achievements are grassroots, wholesome, and empowering, and ensure that trees will not be relegated to a museum.
